# Investment and repayment in a trust game after ventromedial prefrontal damage

**DOI:** 10.3389/fnhum.2013.00593

**Published:** 2013-09-25

**Authors:** Giovanna Moretto, Manuela Sellitto, Giuseppe di Pellegrino

**Affiliations:** Department of Psychology, Centre for Studies and Research in Cognitive Neuroscience, University of BolognaCesena, FC, Italy

**Keywords:** trust, risk, reciprocity, social valuation, vmPFC, lesion studies

## Abstract

Although trust and reciprocity are ubiquitous in social exchange, their neurobiological substrate remains largely unknown. Here, we investigated the effect of damage to the ventromedial prefrontal cortex (vmPFC)—a brain region critical for valuing social information—on individuals’ decisions in a trust game and in a risk game. In the trust game, one player, the investor, is endowed with a sum of money, which she can keep or invest. The amount she decides to invest is tripled and sent to the other player, the trustee, who then decides what fraction to return to the investor. In separate runs, ten patients with focal bilateral damage to the vmPFC and control participants made decision while playing in the role of either investor or trustee with different anonymous counterparts in each run. A risk game was also included in which the investor faced exactly the same decisions as in the trust game, but a random device (i.e., a computer), not another player, determined the final payoffs. Results showed that vmPFC patients’ investments were not modulated by the type of opponent player (e.g., human vs. computer) present in the environment. Thus, vmPFC patients showed comparable risk-taking preferences both in social (trust game) and nonsocial (risk game) contexts. In stark contrast, control participants were less willing to take risk and invest when they believed that they were interacting with people than a computer. Furthermore, when acted as trustee, vmPFC patients made lower back transfers toward investors, thereby showing less reciprocity behavior. Taken together, these results indicate that social valuation and emotion subserved by vmPFC have a critical role in trusting and reciprocity decisions. The present findings support the hypothesis that vmPFC damage may impair affective systems specifically designed for mediating social transaction with other individuals.

## Introduction

Trust is an essential ingredient of human exchange (Arrow, 1974); it lubricates social and economic transactions, and has been long recognized as a critical antecedent of cooperative behavior (Ostrom and Walker, 2003). Trust can be defined as one’s willingness to place resources at disposal of another party in situations in which there is uncertainty regarding the other party’s motive, intentions and actions (Mayer et al., 1995; Rousseau et al., 1998). An action that is trusting of another is one that creates the possibility of mutual benefit, if the other person is cooperative. Yet trusting behaviors also imply the risk of injury or loss to oneself if the other person defects. Overriding aversion to such risks is required for trust to emerge (Kosfeld et al., 2005).

Although theoretical work has identified a number of factors likely to influence trust (Mayer et al., 1995; Lewicki and Wiethoff, 2000), fundamental questions remain about how trust actually operates. For instance, while a commonly held view suggests that trust is a result of rational calculation and higher cognitive processes (Coleman, 1990), in some accounts trust is held to be founded in emotional processes (Hardin, 2002; Butler et al., 2003). Consistent with this latter account, behavioral studies suggest that incidental emotions significantly influence social exchange and trust (Dunn and Schweitzer, 2005). Moreover, several neuroimaging studies have shown that tasks that require social valuation (Winston et al., 2002; Somerville et al., 2006), or cooperation with another individual (McCabe et al., 2001; Gallagher et al., 2002; Rilling et al., 2002, 2004; Tomlin et al., 2006) activate brain regions known to process emotions, including the anterior cingulate cortex and adjacent medial frontal cortex. Importantly, when subjects interact with partners they know to be just computers, these activations are not seen, suggesting that they reflect the interpersonal nature of the task (McCabe et al., 2001; Rilling et al., 2004; Tomlin et al., 2006; van den Bos et al., 2007). Neuroimaging studies, however, do not settle whether a given brain region is necessary for a particular behavior. This issue could be addressed by studying human subjects with focal brain damage. Remarkably, however, only few studies provided causal evidence linking brain areas integral to emotional processes to trusting behavior (van Honk et al., 2012).

Here, we examined whether emotions, specifically social emotions subserved by the ventromedial prefrontal cortex (vmPFC), affect people’s willingness to trust others. Several evidence suggests this possibility. First, the vmPFC is densely interconnected with basolateral amygdala, ventral striatum, and subcortical structures that control autonomic and visceral responses (Carmichael and Price, 1995; Haber et al., 2006), and is therefore ideally located for generating emotional responses, and guiding social interactions. Second, neuroimaging studies in humans have implicated the vmPFC in guiding behavioral choice under uncertainty (Hsu et al., 2005; De Martino et al., 2006), and have argued that this region is critical for balancing potential gains against losses to ensure optimal decision-making in social context (De Quervain et al., 2004). Finally, damage to the vmPFC in humans can be associated with strikingly poor judgment and decision-making (Eslinger and Damasio, 1985; Bechara et al., 1994, 1997; Koenigs et al., 2007), due to markedly reduced (Ciaramelli et al., 2007; Koenigs et al., 2007; Krajbich et al., 2009; Moretto et al., 2009), or poorly regulated (Koenigs and Tranel, 2007) emotions.

To address whether the vmPFC plays a necessary role in the decision to trust a stranger, a sample of patients with adult-onset vmPFC lesions, as well as healthy control subjects (HC) and patients with lesions outside the frontal lobe (non-FC patients), played in the role of investor in a one-round trust/investment game (Berg et al., 1995). This game involves real monetary exchanges between two anonymous individuals, the investor and the trustee, who receive each a sum of money from the experimenter. The investor can keep all the money or decide to invest some amount, which is tripled by the experimenter and sent to the trustee. Next, the trustee decides how much of the tripled amount to return. Money sent by the investor is used to measure her trust, while money returned by the trustee is used to measure her trustworthiness.

Clearly, the decision to trust entails a risk (Rousseau et al., 1998). Uncertainty regarding whether the trustee intends to and will honor the investor’s trust is the source of risk. This raises the important concern over whether a person’s attitude toward general risk influences trust (Eckel and Wilson, 2004; Karlan, 2005; Schechter, 2007). To control for between-group differences in risk attitudes, we therefore also implemented a risk game offering the same options and payoffs as the trust game, but in which a random device (e.g., a computer), not a human partner, determined the investor’s risk. The risk game constitutes a critical control condition because recent behavioral (Bohnet and Zeckhauser, 2004; Hong and Bohnet, 2007; Bohnet et al., 2008; Houser et al., 2009) and neurobiological (Kosfeld et al., 2005; Baumgartner et al., 2008) evidence strongly indicates that the decision to trust is not only determined by risk aversion (i.e., the negative emotion associated with the possibility of losing objects or money) but also by betrayal aversion, that is, the fear to be betrayed by another in social exchange. Betrayal aversion plays no role in the risk game, since random devices are incapable of intentionality or awareness, and they cannot really betray our trust. Therefore, the contrast between trust game and risk game is ideal to assess whether vmPFC damage specifically affects trusting behavior in social exchanges (rather than risk-taking behavior in general), because—except for the type of opponent partner (human vs. computerized partner)—everything else remains constant across these two games. Based on previous findings showing that regions in the vmPFC may be critical for valuing social information (Amodio and Frith, 2006), particularly when the implications of another individual’s intentions must be taken into account before acting (Rudebeck et al., 2008; Behrens et al., 2009; Moretti et al., 2009; Ciaramelli et al., 2013), we hypothesized that investors in the vmPFC-lesioned group would show higher money transfers than those in the control groups, especially in the trust game in which both social and non-social risks operate to inhibit trust.

Several researchers (Andreoni and Miller, 2002; Cox, 2004) have argued that measures of trust taken from the trust game do not discriminate between actions motivated by trust and actions motivated by altruism or generosity. To address this question, we measured the amount of money participants returned when they played the role of trustee in a separate session. If lesion to the vmPFC increases generosity rather than trusting behavior, then one might hypothesize that a player will send more as investor and return more as trustee, thus appearing both more trusting and trustworthy.

Finally, we included a measure of the investor’s subjective expectation about the trustee’s back transfer at different investment levels. This in order to control whether vmPFC patients apparently trust more because they are more optimistic about the trustee’s trustworthiness (e.g., they have higher expected back transfers).

## Materials and Methods

### Participants

Three groups of subjects participated in the study: (a) a group of patients with focal lesions involving the vmPFC (the vmPFC group, *n* = 10), (b) a control group of patients with damage sparing the frontal cortex (the non-FC group, *n* = 10), and (c) a control group of healthy subjects (the HC group, *n* = 10), who were matched on age, education and sex with the vmPFC group. Brain-damaged patients were recruited from the Centre for Studies and Research in Cognitive Neuroscience in Cesena. They were selected on the basis of the location of their lesion evident on computerized tomography (CT) or magnetic resonance imaging (MRI) scans.

Table 1 shows demographic and clinical data, as well as the Mini-Mental Status Examination score (MMSE; Folstein et al., 1983). There were no significant differences between vmPFC patients and comparison groups with regard to age, education, and clinical variables (*p* > .05 in all cases).

**Table 1:** **Summary data for participants [mean (standard deviation)]**.

**Group**	**Sex (M/F)**	**Age at test (year)**	**Education (year)**	**Time since lesion (year)**	**Lesion volume (cc)**	**MMSE**
vmPFC (*n* = 10)	7/3	57.8 (6.6)	10.4 (4.5)	4.6 (2.8)	32.6 (19)	27.1 (2)
non-FC (*n* = 10)	7/3	54 (13.4)	10.3 (3.9)	3.8 (3.5)	26.5 (11.4)	26.3 (1.5)
HC (*n* = 10)	7/3	57.3 (7.3)	9.5 (4.2)	–	–	28 (1.8)

MMSE = Mini-Mental State Examination.

In the vmPFC group, lesions principally involved the vmPFC, which is defined as the medial one-third of the orbital surface and the ventral one-third of the medial surface of the frontal lobe, following the boundaries laid out by Stuss and Levine (2002). Lesion etiology was hemorrhage due to ruptured aneurysm of the anterior communicating artery in 9 out of 10 vmPFC patients, and to traumatic brain injury in one. The vmPFC damage was bilateral (although often asymmetrically so) in six cases, right unilateral in two cases, and left unilateral in two cases. All vmPFC patients presented with clinical evidence of a decline in social interpersonal conduct, impaired decision-making and emotional functioning, but had generally intact intellectual abilities (see Table 2).

**Table 2:** **Results of selected neuropsychological tests [mean (standard deviation)]**.

**Group**	**SRM**	**Digit span forward**	**Phonemic fluency**	**Semantic fluency**	**Corsi**	**Stroop task errors**	**ITS**	**PNR**
vmPFC	35.5 (13)	5 (0.8)	20.2 (9.3)	36.6 (14)	3.7 (0.2)	6.5 (7.3)	2.2 (0.5)	2.9 (1.5)
non-FC	30.6 (4.8)	4 (0.9)	28.2 (10)	42.8 (15)	4.2 (0.7)	1 (1.4)	1.9 (0.4)	2.9 (1.1)
HC	32.2 (3.4)	5.7 (1)	29.2 (9.2)	49.5 (18)	4.8 (0.7)	0 (0)	1.9 (0.4)	2.9 (1.2)

SRM = Standard Raven Matrices (scores in percentile values); ITS = Interpersonal Trust Scale; PNR = Personal norm of Reciprocity scale.

The non-FC patients were selected on the basis of having damage that did not involve the mesial orbital/vmPFC and frontal pole, and also spared the amygdala in both hemispheres. In this group, lesions were unilateral in nine patients (in the left hemisphere in five cases, and in the right hemisphere in four cases) and bilateral in one patient, and were caused by ischemic or hemorrhage stroke in nine cases, and by traumatic brain injury in one case. In the non-FC group, lesion sites included the lateral aspect of the temporal lobe in six patients, the lateral occipital area in two patients, and the occipito-parietal junction in the remaining two patients.

Normal participants were healthy volunteers who were not taking psychoactive medication, and were free of current or past psychiatric or neurological illness as determined by history.

All subject groups were administered a short neuropsychological battery including tests with potential sensitivity to frontal damage, as well as intelligence and memory tests (results are provided in Table 2). The groups differed significantly only in their performance on the Stroop task, with vmPFC subjects making more errors than both non-FC patients and HCs (Mann–Whitney U-test, *p* < .05). Patients were not receiving psychoactive drugs at the time of testing, and had no other diagnosis likely to affect cognition or interfere with participation in the study (e.g., significant psychiatric disease, alcohol misuse, history of cerebrovascular disease, focal neurological examination). Neuropsychological and experimental studies were all conducted in the chronic phase of recovery, more than a year post-onset. All lesions were acquired in adulthood. Patients gave informed consent to participate in the study according to the Declaration of Helsinki (International Committee of Medical Journal Editors, 1991) and the Ethical Committee of the Department of Psychology, University of Bologna.

### Lesion analysis

**Figure 1:**
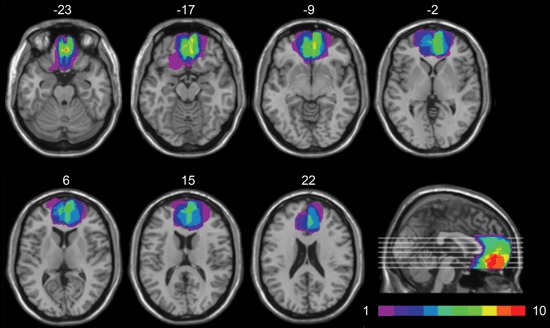
**Location and overlap of brain lesions.** The panel shows the lesions of the 10 patients with vmPFC damage superimposed on the same seven axial slices and on the mesial view of the standard MNI brain. The level of the axial slices has been marked by white horizontal lines on the mesial view of the brain. z-coordinates of each axial slice are given. The color bar indicates the number of overlapping lesions. In each axial slice, the left hemisphere is on the left side.

Lesion analysis was based on the most recent clinical CT or MRI. The location and extent of each lesion were mapped by using MRIcro software (Rorden and Brett, 2000). The lesions were manually drawn by a neurologist with experience in image analysis onto standard brain template from the Montreal Neurological Institute (MNI), which is based on T1-weighted MRI scans, normalized to Talairach space. This scan is distributed with SPM99 and has become a popular template for normalization in functional brain imaging. For superimposing of the individual brain lesions, the same MRIcro software was used. Figure 1 shows the extent and overlap of the brain lesions in the brain-damaged patients. Brodmann’s areas (BA) affected in vmPFC group were areas 10, 11, 12, 32 (subgenual portion), and 24, with region of maximal overlap occurring in BA 10 and 11.

### Experimental design and procedures

Every participant in the experiment played the role of investor in two treatment conditions: a trust game and a risk game. In the trust game, the subject played a standard trust game and she knew her counterpart was human; we call this the human interaction treatment. In the risk game, the subject knew her counterpart was a computer making random decisions; we call this the computer interaction treatment. Trust and risk games were played in separate sessions with an interval of at least 1 week between them. Half of the participants in each group played the trust game in the first session, and half the risk game in the first session.

All experiments took place in a quiet room in which an opaque, removable partition wall was used to create two separate settings. On either side of the wall, we placed a desk with a computer. Participants sat at one desk in front of the computer, while at the other desk sat either an actor who played in the role of the trustee (trust game), or no one (risk game). As a result, playing partners could be separated visually, thereby providing between-subject anonymity, without separating them audibly, thus lending our set-up credibility. Before each session, instructions about the nature and rules of the game were presented on the computer, and the experimenter verbalized them to ensure that participants understood them. In the instructions, it was emphasized that participants in the trust game would play the game anonymously and only once with each opponent player, and that they would receive the money earned in the game. Differently, in the risk game it was emphasized that participants would play with a computer counterpart. After reading the instructions, subjects were required to complete a quiz that required them to state the amount of money that each player would receive under various hypothetical circumstances. The game started once the subject successfully finished the quiz.

Subjects in the role of the investor received no feedback about their partner’s decision between the different interactions. At the end of each session, the experimenter put the cash payoff earned by subject during the game into an opaque envelope that was sealed and signed by the participant. Earnings envelops were kept by the experimenter between games. Subjects did not receive feedback about the outcome of any game until the end of the experiment in order to avoid income effects and the possibility that current decisions were influenced by an opponent’s previous decisions. All games were paid out at the end.

#### Human interaction treatment

Participants acted as investor in a series of nine rounds of a trust game against nine different anonymous human partners via a computer interface. At the beginning of each round, the actor that played the role of the trustee entered the room and sat at her position. When both investor and trustee were ready, the interaction started. Each round was presented as text through a series of five screens. A 6-s initial screen depicted a silhouette of a human figure and indicated the endowment (E) available for both players in the current round. There were three equiprobable initial E, €6, €9 and €12, presented in random order during the game. The second screen posed the question “How many Euros between 0 and E do you transfer to Participant B?” and remained visible until a response was given. Participants were given the opportunity to send any integer amount from zero to their entire endowment available, and were instructed to indicate their decision by pressing the numeric keys of the computer keyboard. Following the response, a screen indicating the investor’s transfer and the amount received by the trustee (three times the amount invested) was presented for 4 s. Then, a variable 5- to 15-s waiting screen informed that the trustee (Participant B) was deciding how much of the tripled amount to send back. Subjects were informed that Participant B could choose the amount from any integer between zero and the tripled amount they have transferred to her/him. Finally, a screen signaled the end of the round. The trustee went out of the room and after a short break was replaced by another actor to begin the next round. When the trustee was out of the room, the investor was asked about her expectation about the trustee’s back transfer.

#### Computer interaction treatment

 Participants were instructed that they would play nine rounds of a risk game in which a random mechanism determined the outcome of the game. In the risk game, everything was identical to the trust game, except that subjects played against a computerized partner. A silhouette of a computer was displayed in the initial screen to indicate the computer interaction. Participants were informed that, in each round, the computer would randomly choose the amount to transfer back from any number between zero and the tripled amount they have transferred to it.

In a separate session, participants played five rounds of a trust game in the role of trustee against five different anonymous investors via a computer interface. The experimental setup was as before, except that participants were assigned the role of trustee (Participant B), and an endowment of €9 was available for both players in every round. Each new round began with a 6-s initial screen that depicted a silhouette of a human figure and indicated that €9 were available for both players in the current round. Then, a variable 5- to 15-s waiting screen informed that the investor (Participant A) was deciding how much between €0 and €9 to transfer to the trustee (Participant B). Next, a screen indicating the investor’s transfer and the amount received by the trustee was presented for 4 s. The investor’s transfers, X, were predetermined and presented randomly, and included one transfer of each €0, €3, €5, €7 and €9, so that the trustee received €0, €9, €15, €21 and €27, respectively. Then, the question “How many Euros between 0 and 3X do you transfer back to Participant A?” appeared on the screen and remained visible until a response was given. Participants were given the opportunity to send back any integer amount from zero to the tripled amount received, and were instructed to indicate their decision by pressing the numeric keys of the computer keyboard. Following the response, a screen signaled the end of the round. The trustee went out of the room and after a short break was replaced by another actor to begin the next round. Note that participants in all groups faced exactly the same set of investors’ transfers. Thus, behavioral differences across these three groups cannot be attributed to differences in the distribution of investors’ transfers.

#### Questionnaires

Approximately 2 weeks after the experiment, participants also completed three self-report questionnaires that assessed selected personality traits. The Personal Norm of Reciprocity (PNR) scale is a 27-item questionnaire measuring three dimensions (nine items each) of reciprocity (i.e., the propensity to reward those who have behaved nicely and punish those who behaved badly): positive reciprocity, negative reciprocity, and beliefs in reciprocity (Perugini et al., 2003); the Interpersonal Trust Scale (ITS) includes 25 component questions requiring subjects to express their trust expectations across a variety of social situations and with diverse social agents (Rotter, 1967).

## Results

**Figure 2:**
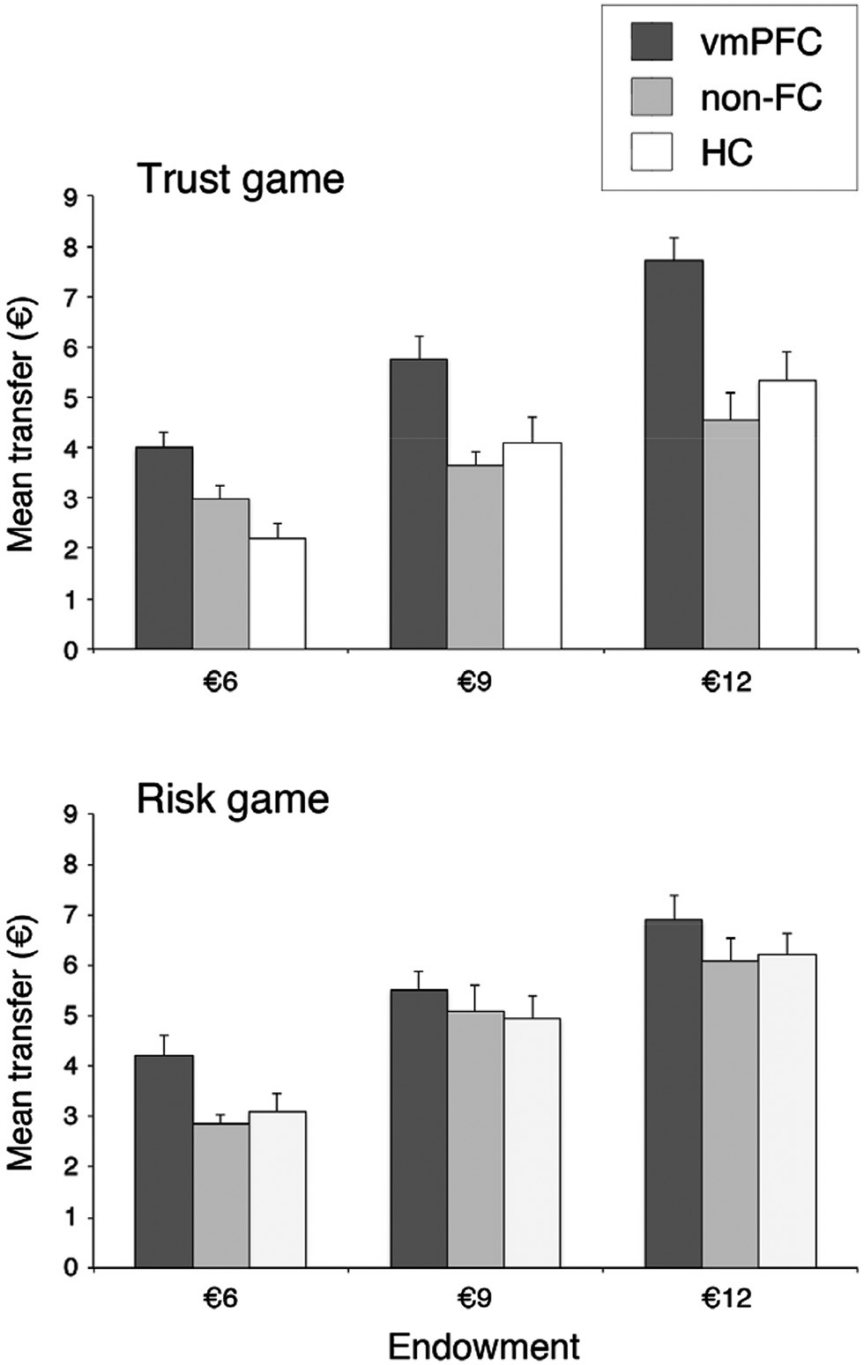
**Groups’ trust level, separately for trust game (upper panel) and risk game (lower panel), and endowment.** Error bars indicate the SEM. vmPFC = ventromedial prefrontal cortex patients; non-FC = control patients; HC = healthy controls.

Figure 2 illustrates investors’ average transfer as a function of initial endowment, separately for the trust and risk game. We performed a mixed design ANOVA on transfer amounts with Group (vmPFC, non-FC, and HC) as a between-subjects factor, and Treatment (human, and computer), and Endowment (€6, €9, and €12) as within-subjects factors. When necessary, pairwise comparisons were conducted using the Fisher LSD test, which is considered the most powerful technique for post hoc tests involving three groups (Cardinal and Aitken, 2006). Analysis showed a significant main effect of Group, *F*(2, 27) = 9.62, *p* < .001, $\eta_{p}^{2}$ = .42, revealing that investors in the vmPFC group had overall significantly higher transfer levels (€5.7 out of a mean endowment of €9) than had investors in the HC (€4.3) and non-FC group (€4.2; both *ps* < .001). There was also a significant main effect of Treatment, *F*(1, 27) = 7.56, *p* < .01, $\eta_{p}^{2}$ = .22, indicating slightly higher transfers in the computer (€5) than in the human (€4.5) interaction, and a significant main effect of Endowment, *F*(2, 54) = 100.14, *p* < .001, $\eta_{p}^{2}$ = .79, demonstrating that investors’ transfer was modulated by initial endowment available.

More critically, analysis showed a significant Treatment by Group interaction, *F*(2, 27) = 4.92, *p* < .02, $\eta_{p}^{2}$ = .27, indicating that the between-group differences in amount sent depended on the human *vs.* computer interaction. Pairwise comparisons showed that when participants played against a human partner, average transfer was significantly higher in the vmPFC group (€5.8) than in both non-FC (€3.7) and HC group (€3.9; both *ps* < .05), while transfers of the control groups did not differ (*p* > .05). By contrast, when participants played against a computerized partner, there was no significant difference between investors’ transfer across groups (vmPFC: €5.5; non-FC: €4.6, HC: €4.7; all *ps* > .05). The identical pattern of results was found when the data were analyzed using nonparametric methods. The Kruskal-Wallis test showed a significant difference amongst the three groups in the trust game (*H* = 12.8, *df* = 2, *p* < .002), but no difference in the risk game (*H* = 4.78, *df* = 2, *p* = .09). Indeed, out of 10 subjects in each group, eight vmPFC patients showed mean transfer levels higher than 50% of initial endowment in the trust game, whereas only three non-FC patients, and four HC displayed such transfers in the trust game. Conversely, in the risk game, nine vmPFC patients, seven non-FC patients, and seven HC displayed mean transfers higher than 50% of initial amount.

The above results suggest that, while control participants decreased their trust level when playing against a human partner as compared to a non-human partner, vmPFC patients failed to modulate their trust based on the recipient of their choices. Thus, damage to vmPFC would lead to an apparent increase in transfer levels in the trust experiment but not in the risk experiment. Accordingly, investors’ transfers in the vmPFC group were not modulated at all by the type of opponent player present in the environment (€5.82 and €5.53, for the trust and risk game, respectively, *p* > .05). In sharp contrast, both control participants were more reluctant to invest in the trust game (€3.71 and €3.88, for non-FC and HC group, respectively), in which interpersonal interactions determines the risk, than in the risk game (€4.69 and €4.74; *p* < .05, and *p* = .01, for non-FC and HC group, respectively), in which a non-social, random mechanism constitutes the risk. This latter result is highly consistent with previous literature in healthy subjects (see De Quervain et al., 2004; Bohnet et al., 2008; Aimone and Houser, 2009; Houser et al., 2009) suggesting that the prospect for betrayal plays a role in trusting decisions well beyond aversion towards monetary loss.

Next, we performed an analysis to explore whether vmPFC patients differed from control groups in their subjective expectations about trustee back transfers in the trust game. To this end, a mixed design ANOVA, with Group (vmPFC, non-FC, and HC) as a between-subjects factor, and Endowment (€6, €9, and €12) as a within-subjects factor, was conducted on expected back transfers divided by the amount sent (a value > 1 indicates expected gain, whereas a value < 1 indicates expected loss from the exchange). Results revealed a significant main effect of Endowment, *F*(2, 54) = 6.70, *p* < .003, $\eta_{p}^{2}$ = .20. More importantly, however, there was no main effect of Group (*F* = 1.42, *p* = .26), nor any interaction between Group and Endowment (*F* = 1.25, *p* = .30), revealing that the three groups of participants believed to obtain on average the same return for their money transferred as investor. Thus, results suggest that the apparent increase in trusting behavior in vmPFC-damaged participants does not depend on subjects’ beliefs about others’ trustworthiness, which was not significantly altered.

**Figure 3:**
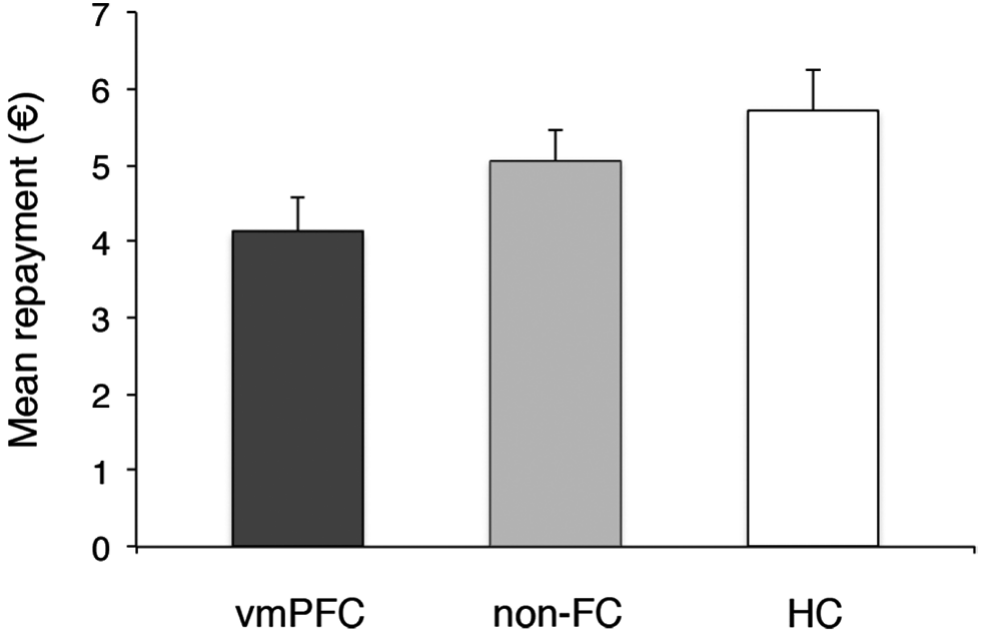
**Group’s reciprocity level.** Error bars indicate the SEM. vmPFC = ventromedial prefrontal cortex patients; non-FC = control patients; HC = healthy controls.

We next tested whether trustees’ repayments to their investor in the trust game differed across the three groups of participants (Figure 3). A one-way ANOVA on trustees’ average back transfers showed a marginally significant effect of Group, *F*(2, 27) = 3.20, *p* = .06, $\eta_{p}^{2}$ = .20. Pairwise comparisons revealed that vmPFC trustees made significantly lower back transfers than HC trustees (mean back transfer: €4.10 and €5.72, for the vmPFC and HC group, respectively, *p* = .02). The non-FC group (mean back transfer: €4.97) was not significantly different from the vmPFC or HC groups (both *ps* > .05), possibly due to higher variance in performance observed in this group. Thus, results indicate that individuals with vmPFC damage do not show more trustworthy or altruistic behavior than control groups.

### Personality questionnaires

Table 2 shows self-report measures of impulsivity, trust, and reciprocity for all three groups of subjects. There were no statistical differences across the three experimental groups on ITS scores (Kruskal-Wallis test, *H* = 4.07, *df* = 2, *p* = .09). Likewise, we found no significant difference amongst the three groups in positive reciprocity scores (Kruskal-Wallis test, *H* = 2.09, *df* = 2, *p* = .35), negative reciprocity scores (Kruskal-Wallis test, *H* = .83, *df* = 2, *p* = .65), and beliefs of reciprocity scores (Kruskal-Wallis test, *H* = .75, *df* = 2, *p* = .69) of the PNR scale.

## Discussion

We show that, following vmPFC damage, economic investments are not modulated by the type of opponent player (e.g., human vs. random mechanism) present in the environment. That is, patients with lesions in the vmPFC showed comparable risk-taking preferences both in social (trust game) and private (risk game) contexts. In stark contrast, control participants were less willing to take risk and invest when they believed that they were interacting with people than a computer (Bohnet and Zeckhauser, 2004). Thus, vmPFC patients invested significantly more than control subjects in the trust game, whilst no difference was observed in the risk game.

These abnormal economic investments were not a general effect of brain damage, because control patients’ behavior was comparable to that of healthy individuals in the trust game, but rather were caused by lesion in a specific prefrontal region vmPFC. Furthermore, the investment of vmPFC-damaged patients in trustees cannot be simply attributed to difference in intellectual, executive or memory abilities, because performance at several neuropsychological tests was similar for vmPFC patients and control participants.

Several mechanisms involved in trusting behavior might be disrupted following damage of vmPFC. One possibility is that vmPFC damage causes a general increase in altruism and prosocial inclinations. On this account, vmPFC damage should affect not only the prosocial behavior of the investors but also that of the trustees. However, the data concerning the trustees’ repayments to their investors in the trust game failed to show more trustworthy or altruistic behavior in the vmPFC group than control groups. On the contrary, data showed reduced generosity in the trustees’ repayment in the vmPFC than in the control groups, thereby indicating that effect of vmPFC damage on trust is not caused by increased generosity or inclination to behave prosocially. This finding is completely consistent with a recent neuropsychological study (Krajbich et al., 2009) demonstrating that vmPFC damage significantly reduces trustworthiness, possibly due to impaired sense of guilt, a sociomoral emotion that plays a critical role also in moral decisions (Ciaramelli et al., 2007; Koenigs et al., 2007; Moretto et al., 2009).

Another possible mechanism behind the effect of vmPFC on trust is that damage to this region alters patients’ subjective expectations or beliefs about others’ trustworthiness or positive reciprocity. In other words, lesion to the vmPFC may render patients more optimistic about the probability of a good return from the investment. However, results showed that these expectations do not differ significantly between vmPFC and control groups, therefore ruling out the possibility that vmPFC patients show more trusting behavior because of unusual or rose-colored beliefs about other players’ repayments. Furthermore, also self-report measures of trust (Rotter, 1967), and reciprocity (PNR, Perugini et al., 2003), indicate that vmPFC patients and control groups hold similar beliefs about others’ trustworthiness and reciprocity. That is, when vmPFC subjects are involved in abstract questions concerning their level of trust or reciprocity they are able to answer not differently from controls groups. This finding is perfectly coherent with results from several other studies (Koenigs et al., 2007; Krajbich et al., 2009; Moretti et al., 2009) showing that an explicit knowledge of social rules, as well as expectations and beliefs are intact and normally accessible following vmPFC damage. Despite this retained knowledge, however, vmPFC patients fail in valuing social information in social interaction and decision-making (Damasio, 1994).

As indicated at the outset, a critical finding of this study emerges when comparing mean investors’ transfer in the trust and the risk games across the three groups of participants. We found, that following vmPFC damage, patients showed higher and similar investments in both games. That is, vmPFC patients did not distinguish between interactions with an intentional agent and those with a computer program that randomly generated outcomes. In striking contrast, control participants were less likely to invest when they believed that they were interacting with people than a computer opponent (Bohnet and Zeckhauser, 2004; Houser et al., 2009), revealing that normal economic decisions are driven by factors beyond mere probability, and that “people care not only about the payoff outcome but also about how the outcome came to be” (Bohnet and Zeckhauser, 2004). Accordingly, trust decisions, relative to risk decisions, entail additional costs, costs shown to be above and beyond mere monetary losses, which diverse authors (Bohnet and Zeckhauser, 2004; Bohnet et al., 2008; Fehr, 2009; Houser et al., 2009) have explained as due to betrayal aversion, namely, the fear to be exploited by others in social interactions. Here, we suggest that, after vmPFC damage, people lack such exploitation aversion, due to impaired social valuation (Amodio and Frith, 2006; Mitchell et al., 2006; Rudebeck et al., 2006; Hare et al., 2010; Tricomi et al., 2010; Zaki et al., 2013), which makes them more willing to take risk arising from interpersonal exchanges. Concerns about “others” do not matter for vmPFC patients, so that they perceive the decision of whether or not to trust basically as a risky choice and decide based on their expectations of trustworthiness and their propensity to risk. That is, it does not matter whether the risk is constituted through the uncertain behavior by the trustee, or through a random mechanism. In this sense, vmPFC patients behave more “rationally” than control participants in our trust game: they only care about their own payoffs and are hardly betrayal averse, as predicted by the standard economic model.

Thus, the seemingly greater level of trust observed in vmPFC patients could be related to their incapacity to value social information and consider negative anticipatory emotional responses related to trusting behavior, specifically they could fail to anticipate in their decision process the value of negative emotional responses associated with the risk of betrayal. Obviously, vmPFC patients’ neglect of potential betrayal and increased willingness to take social risk may invite exploitation and attract selfish actors, which may explain, in part, why their social and financial investments are bound to fail.

A previous study of trust behavior in humans with vmPFC damage failed to find significant difference in economic investment between vmPFC patients and control groups (Krajbich et al., 2009). Several methodological differences may account for the contrasting results between these studies. First, in our trust game choices were continuous and quantitative (e.g., the investor decides how much of her endowment to transfer to the trustee), whereas, in Krajbich et al.’s (2009) study, investor had only binary choices (e.g., trust *vs*. no trust). The binary-choice trust game is easy to implement, but it is less sensitive and likely captures less variation in investor’s trusting behavior. Second, economical exchange with interacting partners was more realistic and salient in the present than in previous study (e.g., their subjects were told that their partners were in another city and were in contact with the experimenter over the phone), which may have also enabled us to find the reported effect. Third, our study involved a larger vmPFC patient sample, which allowed us to reveal a significant difference in trusting behavior after vmPFC damage.

Furthermore, our findings are completely in line with recent evidence of increased rate of investment during a trust game, but not during a risk game, in participants with selective basolateral amygdala damage (van Honk et al., 2012), a region heavily interconnected with the vmPFC (Koolhaas et al., 1990; Bachevalier and Loveland, 2006). The amygdala and vmPFC are thought to act closely together as a part of the neural circuitry regulating many goal-directed behaviors (Murray and Izquierdo, 2007), thereby allowing the selection of advantageous actions in the face of various competing behavioral options. Interestingly, Bos et al. (2010) found decreased trustworthiness in women after being administered testosterone, a hormone targeting on the amygdala. As suggested by Johnson and Breedlove (2010), testosterone might reduce interpersonal trust by acting on neurons in the amygdala to increase communication to systems enabling fearful responses, while reducing communication to orbitofrontal cortex, whereas oxytocin might boost interpersonal trust (see Kosfeld et al., 2005), acting on the same systems with opposite effects.

Thus, previous and current findings suggest that (basolateral) amygdala and vmPFC are critically involved in social economic decisions. Note, however, that several findings from animal studies (see Murray and Izquierdo, 2007, for a review) suggest that, although amygdala and vmPFC functionally interact in mediating some types of adaptive choices, they make distinct contributions to emotional responses and reward processing. For example, while the greater level of trust after basolateral amygdala damage has been interpreted in terms of pathological altruism and generosity (van Honk et al., 2012), the reduced trustworthiness observed in current and previous study (Krajbich et al., 2009) shows such a view to be untenable for vmPFC-lesioned patients. Further research will be necessary to specify the nature of the interaction between the vmPFC and amygdala and how dysfunctions in this circuit differentially contribute to economic decisions in a social context.

Altogether, the above evidence suggests that vmPFC patients, as well as amygdala-lesioned patients, might lack of a mechanism of social vigilance, that is, they could be impaired in the recruitment of social emotions that need to be anticipated correctly in order for decisions to be made optimally. vmPFC, deemed as tuned to the evaluation of social information (Amodio and Frith, 2006), might fail in the recollection of past emotions related to a specific decision by upregulating the value/consequences of future options based on the resulting affective states (Bechara, 2005). However, another mechanism that might be impaired in vmPFC patients is prospection. Prospection refers to the ability to self-project in time (also referred to as mental time travel) to pre-experience future events (Buckner and Carroll, 2007). An impaired prospection might result in myopic, impulsive behaviors. Shortsighted decision-making is indeed a peculiar outcome of vmPFC disruption, resulting in increased impulsive behavior during intertemporal choice (Sellitto et al., 2010, 2011) in increased willingness to judge as acceptable personal violations (Ciaramelli et al., 2007; Moretto et al., 2009; Ciaramelli and di Pellegrino, 2011); in reduced acceptance rate of unfair offers from a human partner (when monetary gains were presented as abstract amounts to be received later) (Moretti et al., 2009); in reduced interpersonal disgust (Ciaramelli et al., 2013). Indeed, the large investments of vmPFC patients in the trust game can be considered shortsighted, impulsive decisions (see also van Honk et al., 2012, for a similar argument).

Taken together, the reported findings allow us to suggest that a lesion in the vmPFC might impair the strategic planning and anticipation of consequences of future events, by both disrupting the correct anticipation of emotions (social emotion, in the current case) to assign them a value, and preventing the optimal construction of possible scenarios following the choice.

In conclusion, these data showed that vmPFC has a critical role in trusting decisions and, in general, is essential for the normal valuation of social stimuli during an economic exchange with another person. These findings are highly compatible with current theories maintaining that vmPFC is a critical neural substrate for forecasting the (positive and negative) emotional consequences of available options in order to guide future behavior, both in personal and societal decision-making (Bechara and Damasio, 2005). Finally, the reported findings provide evidence for theoretical approaches to social cognition and decision-making that emphasize the pivotal role of medial prefrontal cortex in the integration of multiple signals to generate adaptive behavior (Montague and Berns, 2002).

## Conflict of interest statement

The authors declare that the research was conducted in the absence of any commercial or financial relationships that could be construed as a potential conflict of interest.
